# Astrocyte production of the chemokine macrophage inflammatory protein-2 is inhibited by the spice principle curcumin at the level of gene transcription

**DOI:** 10.1186/1742-2094-2-8

**Published:** 2005-02-25

**Authors:** Michiyo Tomita, Brita J Holman, Christopher P Santoro, Thomas J Santoro

**Affiliations:** 1 Department of Medicine, University of North Dakota School of Medicine & Health Sciences, 501 North Columbia Road, Grand Forks, ND 58201, USA; 2 Boston University, 140 Commonwealth Avenue, Chestnut Hill, MA 02467, USA; 3 Loyola University – Chicago, 6525 North Sheridan Road, Chicago, IL 60626, USA; 4 Research Service, Fargo VA Medical Center, 2101 Elm Street, Fargo, ND 58102, USA

**Keywords:** MIP-2, astrocytes, curcumin, gene transcription, chemokines, inflammation

## Abstract

**Background:**

In neuropathological processes associated with neutrophilic infiltrates, such as experimental allergic encephalitis and traumatic injury of the brain, the CXC chemokine, macrophage inflammatory protein-2 (MIP-2) is thought to play a pivotal role in the induction and perpetuation of inflammation in the central nervous system (CNS). The origin of MIP-2 in inflammatory disorders of the brain has not been fully defined but astrocytes appear to be a dominant source of this chemokine.

Curcumin is a spice principle in, and constitutes approximately 4 percent of, turmeric. Curcumin's immunomodulating and antioxidant activities suggest that it might be a useful adjunct in the treatment of neurodegenerative illnesses characterized by inflammation. Relatively unexplored, but relevant to its potential therapeutic efficacy in neuroinflammatory syndromes is the effect of curcumin on chemokine production. To examine the possibility that curcumin may influence CNS inflammation by mechanisms distinct from its known anti-oxidant activities, we studied the effect of this spice principle on the synthesis of MIP-2 by astrocytes.

**Methods:**

Primary astrocytes were prepared from neonatal brains of CBA/CaJ mice. The cells were stimulated with lipopolysaccharide in the presence or absence of various amount of curcumin or epigallocatechin gallate. MIP-2 mRNA was analyzed using semi-quantitative PCR and MIP-2 protein production in the culture supernatants was quantified by ELISA. Astrocytes were transfected with a MIP-2 promoter construct, pGL3-MIP-2, and stimulated with lipopolysaccharide in the presence or absence of curcumin.

**Results:**

The induction of MIP-2 gene expression and the production of MIP-2 protein were inhibited by curcumin. Curcumin also inhibited lipopolysaccharide-induced transcription of the MIP-2 promoter reporter gene construct in primary astrocytes. However MIP-2 gene induction by lipopolysaccharide was not inhibited by another anti-oxidant, epigallocatechin gallate.

**Conclusion:**

Our results indicate that curcumin potently inhibits MIP-2 production at the level of gene transcription and offer further support for its potential use in the treatment of inflammatory conditions of the CNS.

## Background

Curcumin (1,7-Bis 94-hydroxy-3-methoxyphenyl)-1,6 heptadiene-3, 5-di-one) is a spice principle in and constitutes approximately 4% of turmeric and is responsible for curry's characteristic yellow color. As is true of other naturally occurring polyphenolic compounds, such as caffeic acid phenyl ester, rosmaric acid and resveratrol, curcumin possesses antioxidant properties which may reduce the production of free radicals and improve cell viability under conditions of enhanced oxidative stress[1,2]. Curcumin also has anti-inflammatory properties which include the capacity to inhibit 5- and 8-lipoxygenases and cyclooxygenases[3,4], is chemopreventive as evidenced by its capacity to abrogate 12-O-tetradecanoylphorbol-13-acetate (TPA)-induced DNA synthesis and tumor promotion in mouse skin[5], antiproliferative as shown by its suppressive effect on the growth of C6 glioma cells[6], and anti-metastatic as suggested by its ability to inhibit angiogenesis *in vivo*[7].

Curcumin's immunomodulating and antioxidant activities suggest that it might be a useful adjunct in the treatment of neurodegenerative illnesses characterized by inflammation such as Alzheimer's disease[8]. Relatively unexplored, but relevant to its potential therapeutic efficacy in neuroinflammatory syndromes is the effect of curcumin on chemokine production. An active role for chemokines has been demonstrated in the pathogenesis of a variety of central nervous system (CNS) disorders accompanied by inflammation. In neuropathological processes associated with neutrophilic infiltrates, such as experimental allergic encephalitis (EAE) and traumatic injury of the brain, the CXC chemokine, macrophage inflammatory protein-2 (MIP-2) appears to play a pivotal role in the induction and perpetuation of inflammation in the brain[9,10]. In EAE, for example, elevated levels of MIP-2 mRNA and protein preceded infiltration of the CNS by polymorphonuclear leukocytes (PMNs). Similarly, in traumatic brain injury, the kinetics of MIP-2 expression paralleled the recruitment of neutrophils to the inflammatory site[10] and, in experimental bacterial meningitis, neutralization of MIP-2 with a monoclonal antibody attenuated infiltration of the CNS with PMNs[11]. The origin of MIP-2 in inflammatory CNS disorders has not been fully defined, but in EAE astrocytes, appear to be the dominant source of this chemokine[9] and are likely to contribute significantly to MIP-2 production in other neuropathological states as well.

To explore the possibility that curcumin may influence CNS inflammation by mechanisms distinct from its antioxidant and known anti-inflammatory activities, we examined the effect of this spice principle on the synthesis of MIP-2 by astrocytes. Our results indicate that curcumin potently inhibits MIP-2 production at the level of gene transcription and offer further support for its potential use in the treatment of inflammatory conditions of the CNS.

## Methods

### Mice

Six to eight-week-old CBA/CaJ mice were purchased from Jackson Laboratories (Bar Harbor, ME) and bred in our animal facility.

### Materials

Curcumin, epigallocatechin (EGCG) and *E. coli *lipopolysaccharide (LPS; O55B1) were purchased from Sigma, (St Louis, MO). Rabbit anti-cow glial fibrillary acidic protein polyclonal antibody was obtained from Dako Corp. (Carpinteria, CA).

Preparation and culture of astrocytes: Astrocytes were prepared from the brains of neonatal (3 to 7-day-old) CBA/CaJ mice by a modification of the method of Pousset et al[12]. Briefly, four brains were combined, homogenized in 0.25% trypsin through a sterile screen (pore size; 100 μM), incubated for 5 min at 37°C and centrifuged at 400 × *g*. The pellet was suspended in Hank's Buffered Salt Solution (HBSS) and debris was removed by gravity sedimentation on ice for 3 min. The supernatant was collected, centrifuged and the pellet was washed twice with culture medium consisting of DMEM containing 10% heat-inactivated fetal bovine serum (Hyclone, Logan, UT), 1 mM L-glutamate and penicillin/streptomycin (Gibco BRL, Grand Island, NY). The cells were plated on 35 mm dishes and cultured at 37°C in a humidified atmosphere contain 5% C02. After 16 hours, plates were washed to remove non-adherent cells and debris. For experiments in which mRNA or MIP-2 protein were quantified, adherent cells were cultured until they reached confluence. For transfection experiments, adherent cells were cultured until they were nearly confluent. Medium was refreshed in all astrocyte cultures every 2–3 days. The preparations were >98% glial fibrillary acidic protein positive, as measured by flow cytometric analyses using a EPICS XL flow cytometer[13].

Cell viability determination: The effect of curcumin on the viability of astrocytes was assessed by measuring cytosolic lactate dehydrogenase (LDH) leakage into the media as detailed earlier[14]. Briefly, astrocytes were incubated with curcumin (10-^4 ^M to 10-^6 ^M) for up to 48 hours, the supernatants were then harvested and LDH was measured by colorimetric assay using a kit from Sigma diagnostics.

mRNA and protein analyses: Confluent cultures of astrocytes were incubated with LPS (10 ηg to 5 μg/ml) for varying periods of time in the presence or absence of curcumin (10-^4 ^M to10-^7 ^M). After 4 hours of culture, cells were harvested and mRNA was isolated as previously reported[14]. MIP-2 mRNA levels were determined using semi-quantitative polymerase chain amplification (PCR) as described earlier[14] using the primers: 5'-TGCCGGCTCCTCAGTGCT-3' (forward) and 5'-GCCTTGCCTTTGTTCAGTATCTTTTG-3' (backward). In other experiments, the effect of EGCG on induced MIP-2 mRNA production was determined by culturing astrocytes with LPS in the presence or absence of varying doses of the catechin (10-^3^M to 10-^4^M). To assess the effect of curcumin on MIP-2 protein production, astrocytes were cultured with LPS in the presence or absence of curcumin (10-^5^M) for 16 hours. Supernatants were then harvested and MIP-2 levels were determined by enzyme linked immunosorbant assay (ELISA; R&D systems, Minneapolis, MN).

**Figure 1:**
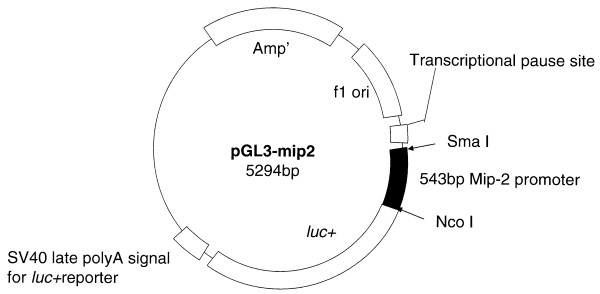
PGL3-MIP-2. A 537 bp fragment, which corresponds to base-pairs -539 to -2 (relative to adenine [assigned +1] in the translation initiation codon of the MIP-2 gene) was generated and inserted into a promotorless luciferase reporter vector, pGL3-Basic.

Preparation of the reporter gene, pGL3-MIP-2: A 537 base pair MIP-2 fragment was prepared by amplifying rat genomic (kidney) DNA using the primers: 5'GCCCACCGAGTCTCTGTTTC3' (forward) and 5'GTTGGTGGCCAGCAGGAGGA3' (backward), then digesting with Rsa I/Nco I. The fragment, which corresponded to base pairs -539 to -2, relative to adenine (assigned +1) in the translation initiation codon of the MIP-2 gene (accession number AJ49888), was ligated to a Sma I/Nco I digested, promoterless luciferase reporter vector, pGL3-Basic (Promega, Madison, WI). The direction of the insert was confirmed by restriction endonuclease digestion and its fidelity determined by sequence analyses as previously described[15]. The MIP-2 promoter-reporter gene construct, pGL3-MIP-2 is shown in Figure 1.

Transfection experiments: Astrocytes were transfected cells using a modification of the method of Franzoso et al[16]. Briefly, 1.5 μg of DNA containing either pGL3-MIP2 or pGL3-basic were incubated in HBS solution (137 mM NaCl, 5 mM KCl, 0.88 mM Na2HPO4, 20 mM Hepes) containing 250 mM CaCl2 for 10 minutes at room temperature. The mixtures were added in 2 mL of media to astrocytes that were nearly confluent. After a 16-hour incubation in a humidified atmosphere at 37 C° containing 5% CO2, cells were washed to remove debris and cultured for an additional 24 hours. LPS plus or minus curcumin (10-^4^M to 10-^7^M) was then added and transfected cells were further cultured for 24 hours. At the conclusion of culture, cells were harvested, cell lysates were prepared, and lysates were analyzed using a luciferase assay system (Promega, Madison, WI) in accordance with the manufacturer's instructions.

## Results and discussion

**Figure 2:**
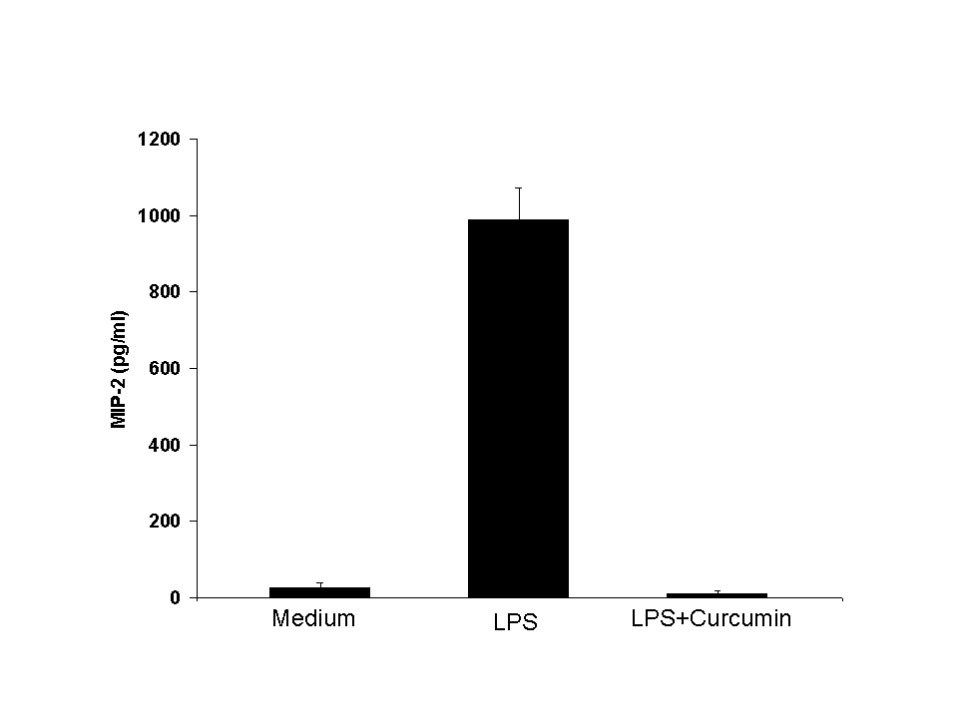
LPS-induced MIP-2 production is inhibited by curcumin. Confluent astrocytes were cultured in medium alone or were stimulated with LPS (5 μg/ml) in the presence (LPS + Curcumin) or absence (LPS) of curcumin (10^-5 ^M). The supernatants were collected after 16 hours and MIP-2 protein (in picograms/ml) was measured by ELISA. Data are the mean ± standard deviation of 4 experiments. Mean MIP-2 production in the medium and LPS + curcumin groups differ significantly from that in the LPS group (p < 0.001 by Student's *t *test). Mean MIP-2 production does not differ significantly between the medium and LPS + curcumin groups (p > 0.2 by Student's *t *test).

To determine whether mechanisms apart from its well-documented anti-oxidant activity might provide possible neuroprotection against inflammation-mediated injury, we investigated the effect of curcumin on astrocyte production of the chemokine MIP-2 in response to LPS. In initial experiments, we found that optimal MIP-2 production occurred when confluent astrocyte cultures were stimulated with 5 μg/ml of LPS during a 16-hour culture (data not shown). Culturing such astrocytes with a dose of curcumin (10-^5^M) that had no effect on viability as measured by LDH release (data not shown), abrogated LPS-stimulated MIP-2 production (Figure 2).

**Figure 3:**
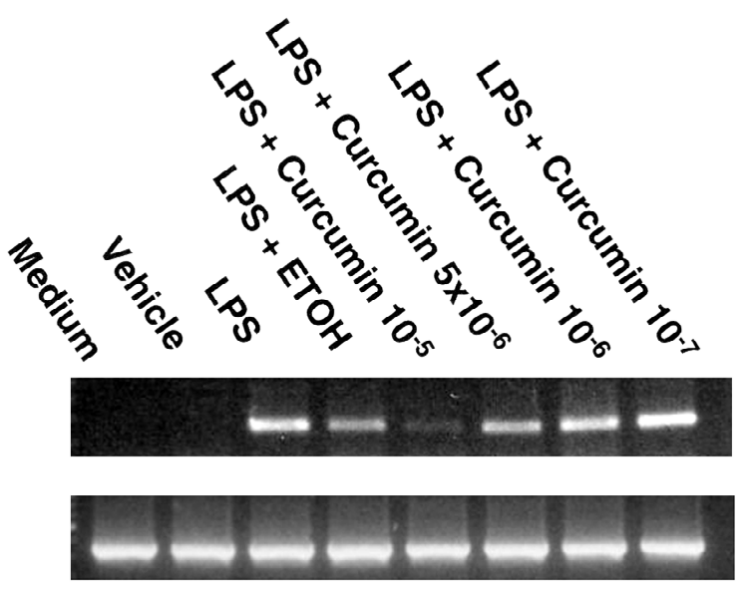
LPS-induced MIP-2 mRNA expression is inhibited by curcumin. Confluent astrocyte cultures were stimulated with LPS (5 μg/ml) in the presence or absence of varying doses of curcumin (10^-5^-10^-7^M) or vehicle (0.05% ethanol), mRNA was then extracted, reverse transcribed and amplified using a mouse MIP-2 primer.

The effect of curcumin on LPS-induced production of MIP-2 mRNA was examined next. Preliminary experiments showed that optimal message for MIP-2 in response to LPS occurred after 4 hours of culture in astrocytes (data not shown). As was true for MIP-2 protein, culture of astrocytes with curcumin (10-^5^M) markedly inhibited chemokine gene expression in response to LPS (Figure 3).

**Figure 4:**
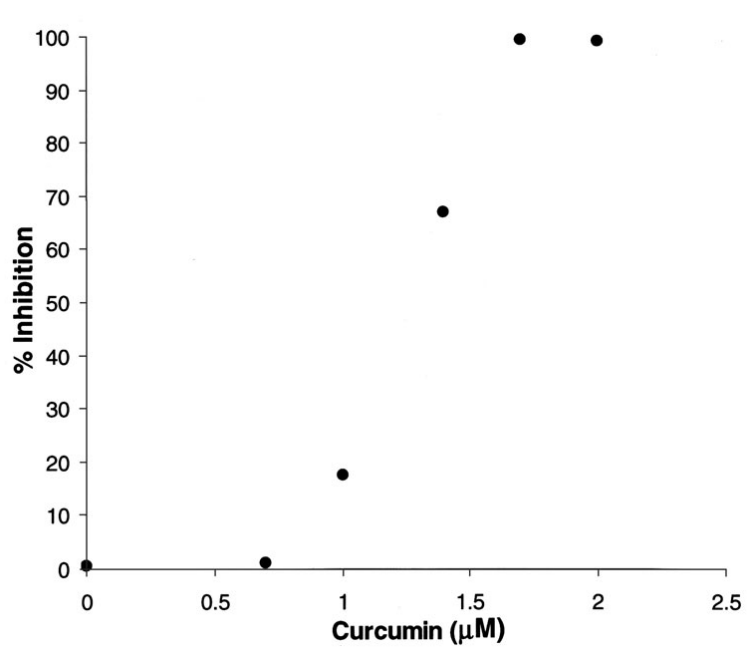
Curcumin inhibits the activity of MIP-2 at the level of gene transcription. Confluent astrocyte cultures were transfected with 1.5 μg of pGL3-MIP-2 or pGL3-Basic and stimulated with LPS (5 μg/ml) in the presence or absence of varying amounts of curcumin (10^-4^-10^-6^M). Transfected cells were harvested and luciferase activity in the cell lysates was quantified. The dose of curcumin is shown in log scale. Results are representative of three experiments.

To determine whether curcumin inhibits MIP-2 gene transcription, a construct was created in which 537 base pairs of the MIP-2 promoter, spanning nucleotides -539 to -2 of the MIP-2 gene (see Methods), were fused to a promoter-less luciferase reporter gene (pGL3-MIP-2, Figure 1). As shown in the representative experiment in Figure 4, curcumin abrogated LPS-stimulated MIP-2 gene expression in transiently transfected astrocytes. In three separate experiments, essentially complete inhibition of LPS-induced MIP-2 gene expression (100%, 92%, 94%) was observed with curcumin in doses of 2 μM.

**Figure 5:**
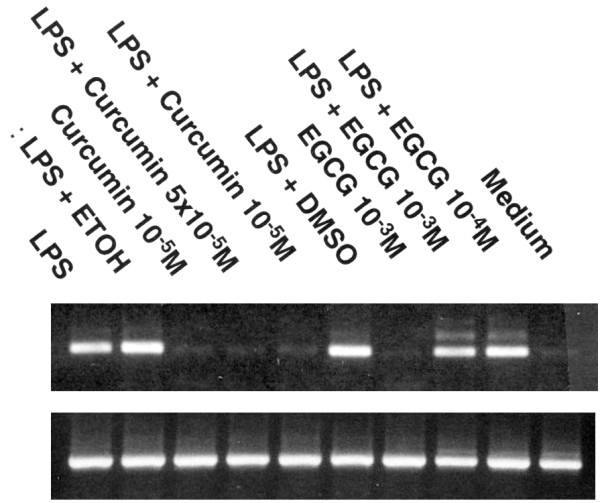
Curcumin but not EGCG inhibits MIP-2 mRNA expression. Confluent cultures of astrocyte were stimulated with LPS (5 μg/ml) in the presence or absence of varying doses of curcumin (10^-5^-10^-7^M), EGCG (10–3), or appropriate vehicle (0.05% ethanol for curcumin, DMSO for EGCG). mRNA was then extracted, reverse transcribed and amplified using mouse MIP-2 primers.

As a specificity control, the effect of EGCG, a catechin present in green tea with potent anti-oxidant activity, was examined on MIP-2 gene expression in astrocytes. In contrast to curcumin, EGCG in doses as high as 10-^3 ^M had no effect on LPS-stimulated MIP-2 mRNA expression (Figure 5). The results suggest that the inhibitory effect of curcumin on MIP-2 production may not be due to its anti-oxidant properties.

The study presented herein shows for the first time that curcumin is a potent inhibitor of inducible MIP-2 production by astrocytes, which are a major source of this chemokine in the brain[9]. In transient transfection experiments of astrocytes, virtually complete inhibition of MIP-2 inducible gene expression was observed with 2 μM curcumin. Since blood levels of curcumin approximating 2 μM were shown by Yang, et al[17] to block amyloid aggregation in a transgenic model of Alzheimer's disease, we believe that our data may have *in vivo *relevance.

Transfection experiments in macrophages using a promoter, reporter-gene construct that contains canonical NFκB and NF-IL-6 cis-acting elements demonstrate that inhibition of MIP-2 by curcumin occurs at the level of gene transcription. The importance of either of these elements in the regulation of inducible MIP-2 gene expression in astrocytes remains to be determined. In some systems, inhibition of NFκB per se by curcumin is sufficient to abrogate gene expression. Thus, curcumin and its hydrogenated metabolites were shown to completely suppress transcription of nitric oxide synthase through down regulation of IκBkinase and NFκB activation in macrophages[18]. However, considering the fact that NFκB activation is linked to multiple upstream signaling pathways[19] and that curcumin has been shown to suppress a number of inflammatory signaling cascades[20], inhibition mediated by this spice principle may be quite complex and highly variable, depending on the cell type and the activating stimulus.

Inhibition of chemokine production represents a novel, potential mechanism by which curcumin may confer neuroprotection in CNS disorders characterized or accompanied by leukocytic infiltration. As stated above, MIP-2 is a dominant, driving force in the pathogenesis of many CNS disorders that are associated with infiltration of neutrophils in the brain[9,10]. Experimentally, recruitment of neutrophils to the CNS is followed by a breeching of the blood-brain barrier that is especially severe after administration of MIP-2[21] and may further contribute to inflammation by causing indiscriminate entry of leukocytes into the brain. The possible contribution of inflammatory infiltrates to neuronal injury is best illustrated by experimental studies in which MIP-2 activity was neutralized. For example, administration of anti-MIP-2 antibody to rats infected with Hemophilus influenza type b abrogated the influx of neutrophils to the meninges, ventricular system, and the periventricular areas of the brain and substantially decreased neuronal damage[11].

In addition to astrocytes, microglial cells and endothelial cells may be potential sources of MIP-2 production in pathological states of the brain. Stimulation of brain microvascular endothelial cells with tumor necrosis factor alpha (TNFα), induces the release of MIP-2 within 4 to 8 hours of *in vitro *culture[22]. Since TNFα levels in the brain are significantly elevated in traumatic brain injury (TBI), it remains possible that cytokine-mediated release of MIP-2 by endothelial cells, particularly those which comprise the blood brain barrier, may predispose to intracerebral neutrophil accumulation and neuronal injury in TBI. Similarly, in a model of hypoxia/reoxygenation, large increases in MIP-2 mRNA and protein were demonstrated in microglial cells suggesting a possible mechanism to account for PMN accumulation and inflammation in cerebral ischemia.

Apart from its ability to inhibit MIP-2 production, curcumin's pleotropic antiinflammatory and anti-oxidative properties suggest its possible use in diseases of the brain accompanied by inflammation. Thus, LPS stimulation transcriptionally upregulates inducible nitric oxide synthase and cyclooxygenase-2 genes in microglia. This leads to the synthesis of nitric oxide (NO) and prostaglandins (PGs), respectively, and the possible formation of neuron-damaging free radicals, such as peroxynitrite. Curcumin abrogates the production of both NO and PGs in LPS activated microglial cells[20]. In a recently completed Phase I clinical trial, oral curcumin at a daily dose of 3.6 grams was, in general, well-tolerated and decreased inducible PGE_2 _production in blood samples taken 1 hour after dose on days 1 and 29 of treatment by approximately 60%[23]. Consistent with its possible use in neurodegenerative diseases associated with oxidative stress injury, curcumin has been reported to decrease oxidative damage and amyloid deposition in a transgenic mouse model of Alzheimer's disease[24], and to reverse Aβ-induced cognitive deficits and neuropathology in rats[25].

In summary, the capacity of curcumin to inhibit astrocyte production of MIP-2, together with its broad immunosuppressive activities, strongly support the potential use of this spice principle in the treatment of inflammatory diseases of the CNS.

## List of abbreviations

EAE, experimental allergic encephalitis; EGCG, epigallocatechin gallate; LDH, lactate dehydrogenase; LPS, lipopolysaccharide; MIP-2, macrophage inflammatory protein-2; NFκB, nuclear factor kappa B; NO, nitric oxide; PG, prostaglandin;

pGL3-MIP-2, a reporter gene construct containing the MIP-2 promoter; PMN, polymorphonuclear leukocyte; TBI, traumatic brain injury; TNFα, tumor necrosis factor alpha.

## Competing interests

The author(s) declare that they have no competing interests.

## Authors' contributions

MT participated in experimental design, acquisition of data, supervised all experiments, and carried out isolation of astrocytes, ELISA and transfection assays.

BH isolated and amplified the MIP-2 gene promoter, and generated the MIP-2 promoter construct, pGL3-MIP-2.

CS participated in culture of astrocytes and PCR analysis of MIP-2 gene.

TS conceived of the study, participated in its design, and helped to draft the manuscript.

All authors read and approved the final manuscript.
